# Effects of Acid Mine Drainage on Calcareous Soil Characteristics and *Lolium perenne* L. Germination

**DOI:** 10.3390/ijerph15122742

**Published:** 2018-12-05

**Authors:** Yan Dong, Fenwu Liu, Xingxing Qiao, Lixiang Zhou, Wenlong Bi

**Affiliations:** 1 Environmental Engineering Laboratory, College of Resource and Environment, Shanxi Agricultural University, Taigu 030801, China; dongyan@sxau.edu.cn (Y.D.); qiaoxingxing@stu.sxau.edu.cn (X.Q.); bwl@sxau.edu.cn (W.B.); 2 Department of Environmental Engineering, College of Resources and Environmental Sciences, Nanjing Agricultural University, Nanjing 210095, China; lxzhou@njau.edu.cn

**Keywords:** acid mine drainage (AMD), AMD-polluted soil, soil nutrients, heavy metals, seed germination

## Abstract

Acid mine drainage (AMD) is a serious environmental problem resulting from extensive sulfide mining activities. There is a lack of more comprehensive and detailed studies on the effect of AMD on calcareous soil characteristics and seed germination. In this study, five calcareous soil samples, collected from Xiaoyi, Taigu, Xiangning, Hejin, and Xixian counties in Shanxi Province, China, were used to investigate the effects of acid AMD on soil characteristics and *Lolium perenne* L. germination through laboratory culture experiments. The results showed that the increase in the total soil calcium oxide and magnesium oxide (CaO + MgO) contents led to a rise in the amount of Fe^2+^ in AMD converted into Fe^3+^, and that major ions (H^+^, Fe, SO_4_^2−^) in AMD were trapped in the soil. The total Cao + MgO contents in the soil collected from Hejin and Taigu counties were 14.23% and 6.42%, the pH of AMD-polluted soil decreased to 7.24 and 3.10, and 98.7% and 54.0% of the Fe^2+^, 99.9% and 58.6% of the total Fe, and 76.0% and 26.4% of the SO_4_^2−^, respectively, were trapped in the soil when the AMD volume to soil mass ratio was 10 mL/g. The results for the soil from Taigu County showed that when the soil had an AMD volume to soil mass ratio of 10 mL/g, the organic matter, available phosphorus (available P), available potassium (available K), Cr, and Cd contents in soil decreased by 16.2%, 63.0%, 97.1%, 7.8%, and 73.2%, respectively; the total phosphorus (total P) and total potassium (total K) did not significantly change; whereas the available nitrogen (available N) and total nitrogen (total N) increased to 16.1 times and 1.76 times, respectively. Compared to the initial soil collected from Taigu County, the *Lolium perenne* L. germination rate decreased by 81.1%, and the cumulative amount of Cr in the *Lolium perenne* L. increased by 7.24 times in the AMD-polluted soil when the AMD volume to soil mass ratio was 6 mL/g. The soil conditions could not support *Lolium perenne* L. germination when the AMD volume to soil mass ratio was 10 mL/g. The outcomes of this study could have important implication in understanding the hydrological/geochemical-behaviour of major ions of AMD in calcareous soil. The findings also have great significance in predicting plant growth behavior in AMD-polluted calcareous soil.

## 1. Introduction

Soil is a basic substrate in terrestrial ecosystems and is also the primary basis for agricultural production. Soil pollution has always been an important issue in environmental science and soil science [1,2,3]. China has experienced increasing industrialization and rapid urbanization over the past 30 years, and large amounts of chemical pollutants have entered the soil through sewage irrigation, mining, and the storage of solid waste [4,5].

Acid mine drainage (AMD) production, due to extensive sulfide mining activities, is a serious environmental problem [6]. During mining, large amounts of sulfide minerals, such as pyrite ore (FeS_2_), are exposed to air, water, and micro-bacteria, and eventually generate AMD [7]. AMD is a special type of acidic drainage that has a low pH and contains high levels of Fe, SO_4_^2−^, and heavy metals. These characteristics contribute to both surface water pollution and soil pollution [8]. Soil acidification, soil heavy metals pollution, soil iron and sulfate pollution, and crop health in AMD-polluted soil environments are critical issues that have been investigated across the world [9,10,11]. Moreover, Fe, S cycling and Fe and S mineral phase transformation regulating metalloids mobility in acid sulfate soil environment have also been given more attention in the world [12,13].

In China, soil pollution by AMD has mainly focused on the AMD-polluted paddy red soil around the Dabaoshan mine area in Guangdong Province. Qu et al. [14] investigated the heavy metal contents in AMD-polluted paddy soil samples from the Dabaoshan mine area that had pHs of 4.34–6.49. They found that Cu, Zn, and Cd levels in the topsoil were, respectively, 2.5–7.5, 1.0–2.1, and 2.2–5.5 times higher than the maximum allowable levels for Chinese agricultural soil [15]. Non-residual forms of Cu, Zn, and Cd in such AMD-polluted paddy soil samples accounted for 29.20–74.41%, 21.69–45.02%, and 58.05–93.65% of their total concentrations, respectively. Li et al. [16] reported that Cd and Zn were preferentially leached from AMD-polluted paddy soil in the Dabaoshan mine area and had higher solubilities than Cu and Pb when the soil pH ranged from 3.0–5.0. In the AMD-polluted paddy soil, the largest concentrations of adsorbed sulfate were found in the 0.2–0.3 m soil horizon where large surface area soil particles, abundant amorphous iron hydroxides, and fine clays enhance SO_4_^2−^ adsorption [17]. In this mining area, Liao et al. [18] collected and analyzed 28 sugarcane, 30 vegetable, and 16 paddy rice crop samples for heavy metals and reported that Cd and Zn uptake by roots contributed to their high concentrations in crops. This was probably due to the high exchangeable fraction levels in the soil. The metal concentrations in sugarcane roots were higher than in the rice and vegetable roots.

The AMD problem in Shanxi Province, China, has also attracted attention in recent years [19,20] because nearly a quarter of Chinese coal resources are distributed in this province. Rare earth element (REE) concentrations in AMD from the Sitai and the Malan coal mines in Shanxi Province have been determined and the results showed that pH is the most important factor controlling REE fractionation [20]. In addition, SO_4_^2−^ complexes (LnSO_4_^+^ > 60%) and free metal species (Ln^3+^, 20–40%) are dominant REEs species in the AMD from the Sitai coal mine [19]. In contrast to red soils in Guangdong Province, typical soil in Shanxi Province is a calcareous soil [21]. Unfortunately, the study of calcareous soil contamination by AMD has not attracted much attention in Shanxi Province. In our previous study, the migration behavior of the major ions from AMD affected calcareous soil was investigated and the results showed that almost all Fe ions (>99%) from the AMD and >80% of SO_4_^2−^ were retained within 0–20 cm soil layer [21]. However, acid buffering properties of the soil collected from the different Shanxi regions against AMD have not yet been revealed. In addition, the soil nutrient and heavy metals content change when calcareous soil is polluted by AMD, and the influence of AMD-contaminated soil on grass seed germination still needs further study.

In view of this, the main objectives of this study were to: (1) investigate the effect of AMD on calcareous soil characteristics, such as explore the interface behavior of the major ions of AMD (H^+^, total Fe, Fe^2+^, and SO_4_^2−^) in soil and AMD, and investigate the change of soil nutrients and heavy metals oxidation state after AMD enters the calcareous soil; and (2) identify the effects of AMD-contaminated soil on grass (*Lolium perenne* L.) seed germination.

## 2. Materials and Methods 

### 2.1. Soil Sampling

**Figure 1:**
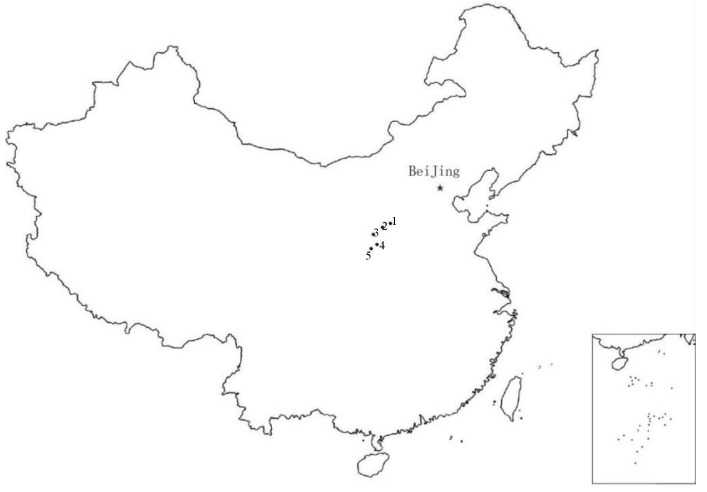
The location of soil sampling sites in China (soil samples 1, 2, 3, 4, and 5 represent the soil samples collected from Taigu County, Xiaoyi County, Xixian County, Xiangning County, and Hejin County).

Five soil samples were collected from agricultural fields at five sites located in Xiaoyi County (city) (111°47′44″ E, 37°10′14” N), Taigu County (112°32′37″ E, 37°24′20″ N), Xiangning County (111°10′15″ E, 35°55′15″ N), Hejin County (city) (110°37′30″ E ,35°39′33″ N), and Xixian County (110°55′49″ E, 36°41′22″ N). In each field plot, three random, separate soil subsamples were taken from 0–0.2 m depth of surface soil within an area of 300 m^2^ and combined into one sample and brought back to the laboratory. Five soil sampling sites are located in the central part of China. What’s more, Xiaoyi County and Taigu County are located in the central part of Shanxi Province. Hejin County, Xiangning County, and Xixian County are located at the southwest part of Shanxi Province. The location of the soil sampling sites in this study are shown in Figure 1. 

The soils were then air-dried at ambient temperature. Some subsamples were ground and sieved to 1 mm to determine the pH, physical clay content, available nitrogen (available N), available phosphorus (available P), and available potassium (available K). Some subsamples were ground and sieved to 0.154 mm and then used to determine the organic matter, total nitrogen (total N), total phosphorus (total P), and total potassium (total K) contents of the soil using the semi-micro Kjeldahl method [22], NaOH melting and Mo-Sb anti-spectrophotometry method [23], and NaOH melting and flame photometric method [24], respectively. The other subsamples were ground and sieved to 0.074 mm to determine the calcium oxide (CaO) and magnesium oxide (MgO) contents using an X-ray fluorescence spectrometer [25]. The physical and chemical characteristics of the soil samples are shown in Table 1.

### 2.2. Stimulated Acid Mine Drainage (AMD) Preparation

A 25 L bioreactor was used to dissolve 667 g FeSO_4_·7H_2_O in 12 L of deionized water. After FeSO_4_·7H_2_O had completely dissolved, 1.5 L of modified Fe-free 9 K medium stock solution (0.168 g Ca(NO_3_)_2_, 0.58 g K_2_HPO_4_, 1.19 g KCl, 5.83 g MgSO_4_·7H_2_O, and 35 g (NH_4_)_2_SO_4_ in 1 L of deionized H_2_O, adjusted to pH 2.50 with H_2_SO_4_) and 1.5 L of *Acidithiobacillus ferrooxidans* LX5 (CGMCC No.0727) culture medium [26] were added into the system. The aeration rate and temperature in the bioreactor were maintained at 0.055 m^3^/min and ~28 °C, respectively. The ferrous ions were almost completely bio-oxidized after 48 h. Cultures were then filtered through quantitative filter paper and the filtrate was collected as stimulated AMD. Two batches of stimulated AMD were prepared using the above method. The AMD collected from the first batch was used for the experiments outlined in Section 2.3 and Section 2.4, and the pH, total Fe, Fe^2+^, and SO_4_^−^ concentrations in the first batch were 2.03, 8302 mg/L, 118 mg/L, and 23,462 mg/L, respectively. The AMD collected from the second batch was used for the experiments outlined in Section 2.5, and pH, total Fe, Fe^2+^, and SO_4_^2−^ concentrations for the second batch were 2.03, 8079 mg/L, 89 mg/L, and 25,672 mg/L, respectively. 

### 2.3. Interface Behavior of the Major Ions of AMD in Soil and Water after AMD is Added to Calcareous Soil 

In this experiment, 10 g of air-dried soil collected from Xixian County, Xiaoyi County, Xiangning County, Hejin County, and Taigu County in Shanxi Province that had been stored in 250 mL glass Erlenmeyer flasks were combined with 20, 40, 60, 80 or 100 mL of AMD to create AMD volume to soil mass ratios of 2, 4, 6, 8, or 10 mL/g, respectively. The AMD volume to soil mass ratios were selected based on the relevant data obtained in the pre-experiment. All treatments were repeated three times. All the Erlenmeyer flasks were sealed with eight-layer gauze and the AMD was made to react with the soil at 30 °C on a reciprocating shaker set to 180 r/min. After 12 h, the solution in each flask was filtered using double layer filter paper, and the filtrate and AMD-polluted soil were collected. The pH of the AMD-polluted soil and the total Fe, Fe^2+^, and SO_4_^2-^ concentrations in AMD after it had reacted with the soil samples were measured. 

### 2.4. Effects of AMD on Calcareous Soil Characteristics

A total of 100 g of air-dried soil (<1 mm) from Taigu County was placed in 500, 1000, or 2000 mL glass Erlenmeyer flasks and 200, 400, 600, 800, or 1000 mL of AMD was added to the corresponding flasks, respectively, to make an AMD volume to soil mass ratio of 2, 4, 6, 8, or 10 mL/g. All treatments were repeated three times. The solutions were sealed with eight-layer gauze and placed on a reciprocating shaker at 30 °C and 180 r/min. After 12 h, the solution in each flask was filtered and the AMD-polluted soil was collected. The organic matter, total N, total P, total K, available N, available P, available K, and heavy metal (Cr, Cd) contents and states in the AMD-polluted soils were analyzed. 

### 2.5. Effect of AMD-Polluted Calcareous Soil on Lolium Perenne Seed Germination Efficiency

A total of 150 g of soil (<1 mm) from Taigu County was placed in 15 plastic containers and then 0.3 L, and 0.9 L, or 1.5 L of AMD was added the plastic containers. There were five containers for each treatment. The containers were then left at 20–25 °C for six months. After six months, the slurry in each plastic container was filtered using double layer filter paper and the treated soil was air dried, ground, and sieved to 0.154 mm. The soil had reacted with the AMD and the AMD volume to soil mass ratios were 2 mL/g, 6 mL/g, or 10 mL/g, respectively. The treated soil samples were then labeled as AMD_0.3L_-treated soil, AMD_0.9L_-treated soil, or AMD_1.5L_-treated soil. The soil pH and total salinity (total content of water-soluble salt) was then determined.

In the seed germination experiment, the bottoms of 20 clean flowerpots (7 cm diameter, 8 cm in height) were covered with double gauze. A total of 120 g of initial soil, AMD_0.3L_-treated soil, AMD_0.9L_-treated soil, or AMD_1.5L_-treated soil was placed in the flowerpots. The treatments were replicated five times. Then, 50 uniform seeds of *Lolium perenne* L. (Merida variety, Jiangsu Suqian Poultry Industry, Suqian, China) were sown 1 cm below the soil surface in each flowerpot. A total of 40 mL deionized water was added to each flowerpot and then they were cultivated at 25 °C. The number of germinated seeds was recorded every day during the 8-day cultivation process. The seed germination percentage and the height of the *Lolium perenne* L. seedlings were also measured. The germination percentage was defined as the number of germinated seeds/the total number of seeds planted × 100. After cultivation, *Lolium perenne* L. collected from different treatments were washed in deionized water and dried at 105 °C. Then the biomass (dry weight) of each *Lolium perenne* L. seedling and the heavy metal contents were measured. The Cr and Cd were selected as the target heavy metal elements. It should be noted that, due to the limited biomass of *Lolium perenne* L., Cr and Cd contents in *Lolium perenne* L. were analyzed in three replicates.

### 2.6. Analytical Methods

Soil pH was determined using a pHS-3C model digital pH-meter (Shanghai Yueping Instruments Co., Ltd., Shanghai, China) by mixing the soil with 0.01 mol/L CaCl_2_ solution (1:2.5 *v*/*v*) to create a slurry. The pH was determined after shaking the slurry for 15 min [27]. Soil total salinity was measured by a DDS-307+ type conductivity meter (Chengdu Fangzhou Technology Co., Ltd., Chengdu, China) that measured the electrical conductivity of a solution extracted from a water-saturated soil paste [28]. The soil organic matter contents were measured using the oil heating and potassium dichromate-volumetric method [29]. The SO_4_^2−^ in solution was determined using the barium sulfate turbidimetric method [21]. The total Fe and Fe^2+^contents in solution were determined using 1,10-phenanthroline method [30], and Fe^3+^ contents in solution were assumed to be the difference between the total Fe and Fe^2+^ contents. The physical clay content of the soil was determined using the hydrometer method [31]. It is noted that the physical clay in here is delineated according to Katschinski’s system of the former Soviet Union. The available N, available P, and available K in soil were determined by the alkali diffusion method [32], the sodium bicarbonate extraction and Mo-Sb anti-spectrophotometry method [33], and the neutral normal ammonium acetate extraction and flame photometric method [34], respectively. The total N, total P, and total K in the soil were determined using the semi-micro Kjeldahl method [22], NaOH melting and Mo-Sb anti-spectrophotometry method [23], and NaOH melting and flame photometric method [24], respectively. CaO and MgO contents in soil were determined using an X-ray fluorescence spectrometer (ZSX-Primus II, Rigaku, Tokyo, Japan) [25], and the Cr and Cd contents in the soil and *Lolium perenne* L. were measured by a TAS-990 flame atomic absorption spectrometer (Beijing Purkinje General Instrument Co., Ltd., Beijing, China) [35]. The biomass (dry weight) of *Lolium perenne* L. from the different treatments was measured using an electronic balance (Sartorius BS 124S, Beijing, China) at a precision of 0.0001 g. All chemicals used in this research were of analytical grade.

### 2.7. Statistical Analyses

All data in this study were analyzed using Microsoft Excel 2010 (Microsoft Corporation, Redmond, WA, USA) software. The data presented in the Tables and Figures were the mean values with standard deviations. The figures were generated by the Origin 9.0 software (OriginLab Corporation, Northampton, MA, USA). Pearson’s correlations were calculated using SPSS 20 statistical software (IBM Corp., Armonk, NY, USA), and the *F*-test was used to analyze significant differences under significant correlation at *p* < 0.05 level. 

## 3. Results and Discussion

### 3.1. Acid Buffering Properties of the Soil Collected from Different Shanxi Regions 

**Figure 2:**
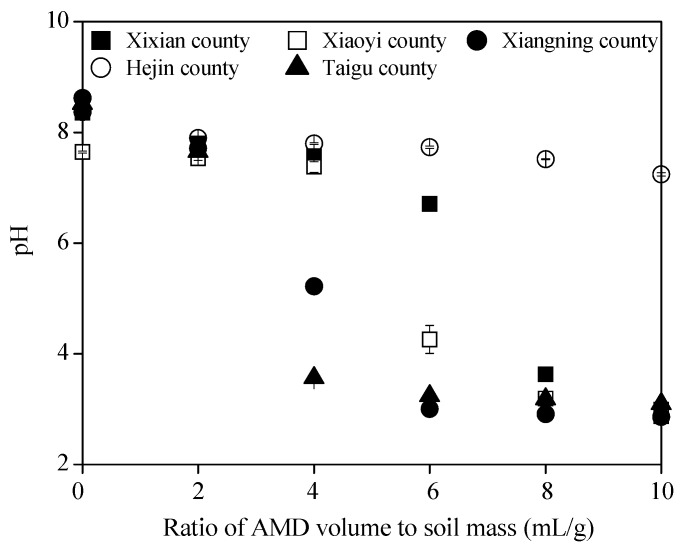
Changes in soil pH after AMD (acid mine drainage) was introduced into the soil (0 on the x axis is the initial soil pH).

The initial pHs of the soils collected from Xixian County, Xiaoxi County, Xiangning County, Hejin County, and Taigu County were 8.36, 7.64, 8.62, 8.37, and 8.52, respectively. The soil pHs decreased to pH ~7.70 when the AMD volume to soil mass ratio was 2 mL/g (Figure 2). 

The pH of the soil collected from Taigu County decreased to 7.65 at an AMD volume to soil mass ratio of 2 mL/g. The pHs decreased to 2.86–3.10 in the Xixian County, Xiaoyi County, Xiangning County, and Taigu County soils when the AMD volume to soil mass ratio was 10 mL/g, but the Hejin County soil pH only decreased to 7.24. This indicated that the acid buffering capacity of calcareous soils from the different Shanxi regions varied. Soil acid buffering capacity is governed mostly by organic matter, clay content, dissolution/precipitation of carbonates, oxides (hydroxides), and ions exchange [36]. In Shanxi Province, the dominant type of soil is calcareous soil, which contains high levels of calcium carbonate and magnesium carbonate. The calcium carbonate content in most of the cultivated soils in Shanxi Province can reach 5–15% [37]. The correlations between soil buffering capacity and organic matter, physical clay content, CaO, and MgO were analyzed in this study. The results showed that soil pH reduction was significantly correlated with the total CaO contents (or calcium carbonates) because the correlation coefficient was −0.906 (Table 2). The soil pH reduction was also significantly correlated with total CaO and MgO contents, with a correlation coefficient of −0.913. Therefore, higher the total CaO (or magnesium carbonate) and MgO (or calcium carbonates) contents in the soil, then higher the buffer capacity of soil to H^+^ from AMD, and smaller the pH reduction when AMD pollutes the soil. It is clear that CaO plays a decisive role in increasing soil acid buffering capacity. The results show that the organic matter and physical clay in the calcareous soil from Shanxi Province have no significant correlation with soil acid buffering capacity (Table 2). Magdoff and Bartlett [38] investigated that soil pH buffering capacity in Vermont, USA, and found that the amount of soil organic matter appeared to play an important role in buffering soil pH. In Vermont, the mean pH value and mean organic matter content were 5.7 ± 0.9 and 4.67% ± 2.17%, respectively [38]. However, the mean pH value and mean organic matter content in this study were 8.30 ± 0.39 and 2.12% ± 0.43%, respectively. Furthermore, CaO content was about 2.28–4.29 times greater than the organic matter content in the collected soils. Therefore, a high CaO (or calcium carbonates) content and a low organic matter content may explain why CaO contribution to the acid buffering capacity of the soil was greater than organic matter in this study.

### 3.2. Total Fe, Fe^2+^, and SO_4_^2−^ Concentrations in AMD when It Reacts with the Calcareous Soil 

**Figure 3:**
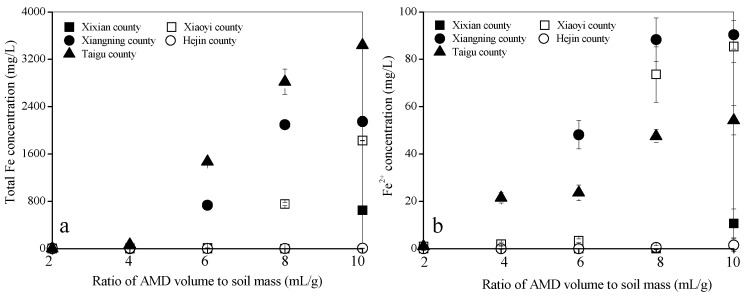
Changes in AMD total Fe (**a**) and Fe^2+^ (**b**) concentrations when it reacts with soils collected from different regions in Shanxi Province.

The total Fe and Fe^2+^ concentrations in AMD were 8302 mg/L and 118 mg/L, respectively. Figure 3 shows the total Fe and Fe^2+^ concentrations in AMD when it reacts with the soil under different treatments. Increasing AMD levels led to a gradual rise in the total Fe concentration in AMD when it reacted with the soil collected from Xixian County, Xiaoyi County, Xiangning County, and Taigu County. A comparison between Figure 2 and Figure 3a shows that the gradual acidification of the soil system is the main factor causing the total Fe concentration to gradually increase in the AMD solution. Soil pH of 3.50 may be the critical pH that determines whether the total Fe remains high in AMD. In other words, when the soil pH is higher than 3.50, the total Fe residue in AMD is very low (<10 mg/L), and when the soil pH is lower than 3.50, the total Fe residue in AMD increases significantly. For example, the total Fe levels in AMD were 3.0 mg/L, 68.6 mg/L, 1471.9 mg/L, 2819.4 mg/L, and 3439.2 mg/L when the AMD reacted with Taigu County soils when AMD volume to soil mass ratios were 2 mL/g, 4 mL/g, 6 mL/g, 8 mL/g, and 10 mL/g, and the soil pH changed to 7.65, 3.57, 3.24, 3.19, and 3.10, respectively. 

These results agreed with Liu et al. [21] who found that the total Fe in AMD almost completely removed when the pH of the AMD-polluted soil was above 3.65. The results for the soil collected from Hejin County showed that the total Fe concentrations in AMD ranged from 4.52–8.53 mg/L when the AMD volume to soil mass ratios were 2–10 mL/g. These low levels were probably due to the AMD-polluted soils having the pHs above 7.00. 

The Fe^2+^ concentrations in AMD when it reacts with the soil in the different treatments are shown in Figure 3b. The Fe^2+^ concentrations in AMD changed from an initial 118 mg/L to 0 mg/L when the AMD volume to soil mass ratio was 2 mL/g and the pH of the different treatments was above 7.50. The results agree with Xu et al. [39] who found that the Fe^2+^ is converted to Fe^3+^ when the pH of the system is above 4.50. They also found that the longer the reaction time, the higher the Fe^2+^ oxidation rate. The Fe^2+^ concentrations in AMD changed to 10.7 mg/L, 85.4 mg/L, 90.3 mg/L, 1.51 mg/L, and 54.3 mg/L when AMD was added to the soils collected from Xi County, Xiaoxi County, Xiangning County, Hejin County, and Taigu County, respectively, and the AMD volume to soil mass ratio was 10 mL/g and pHs of AMD-polluted soil were 2.87, 2.99, 2.86, 7.24, and 3.10, respectively. When a small amount of AMD is added to calcareous soil, Fe^2+^ in the AMD system will be oxidized to Fe^3+^, which means that Fe^2+^ is no longer a typical ions in AMD. However, Fe^2+^ may be a typical ions in AMD after a large amount of AMD reacts with calcareous soil because it may cause the pH of the AMD-polluted soil to drop below 4.50. 

**Figure 4:**
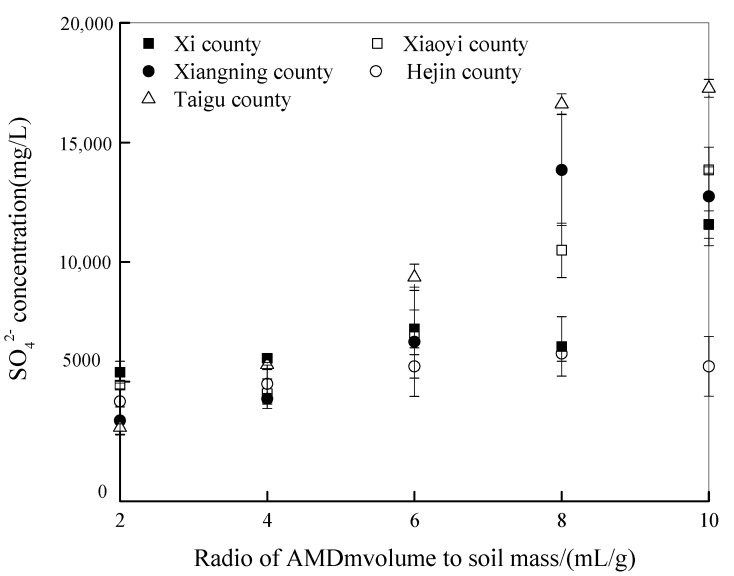
Changes in AMD SO_4_^2−^ concentrations when it reacts with soils collected from different regions in Shanxi Province.

The SO_4_^2−^ concentrations in AMD when it reacts with the soil in the different treatments are shown in Figure 4. The SO_4_^2-^ concentrations in AMD decreased from an initial 23,462 mg/L to 3080–5391 mg/L in the different treatments when the AMD volume to soil mass ratio was 2mL/g. The SO_4_^2−^ concentration in AMD gradually increased as the AMD addition levels rose in the soils collected from Xixian County, Xiaoyi County, Xiangning County, and Taigu County. For example, the SO_4_^2−^ concentration in AMD decreased to 3080 mg/L, 5705 mg/L, 9364 mg/L, 16,607 mg/L, and 17,264 mg/L in the soil collected from Taigu county at AMD volume to soil mass ratios of 2 mL/g, 4 mL/g, 6 mL/g, 8 mL/g, and 10 mL/g, respectively. However, there was no significant change in the AMD SO_4_^2-^ concentration (4173–6173 mg/L) when it was added to the soil collected from Hejin County and the AMD volume to soil mass ratios were between 2 and 10 mL/g. Liu et al. [21] reported that calcium carbonate can be transformed into calcium sulfate when calcareous soil is polluted with AMD. Therefore, CaO and MgO contents in calcareous soils not only determines the acid neutralizing capacity, or acid buffering capacity, of the soil but also determines the chemical behavior of total Fe, Fe^2+^, and SO_4_^2−^ after AMD enters the soil. It can be concluded that in calcareous soil, higher the CaO and MgO levels in the soil, higher the soil pH, and easier it is for Fe^2+^ to be transformed into Fe^3+^ in the soil system. The total Fe and SO_4_^2−^ retained by the soil also increases and there is weaker vertical migration of the major ions (H^+^, Fe, SO_4_^2−^) of AMD.

In addition, Blodau et al. [40] reported that the pH significantly regulates the Fe^2+^ oxidation, minerals dissolution, iron precipitation, iron hydroxide transformation, and iron and sulfate reduction in acid coal mine lakes and their watersheds. Karimian et al. [41] found that the low pH may be a key factor limiting microbially mediated SO_4_^2−^ reduction. It can be seen that the pH of soil system not only affects the precipitation behavior of Fe and S, but also affects the transformation of Fe and S in soil system.

### 3.3. Effects of AMD on Calcareous Soil Characteristics

The initial organic matter, total N, total P, total K, available N, available P, and available K in the soil collected from Taigu County were 18.32, 0.93, 0.68, 26.94, 30.71, 18.45, and 218.23 mg/kg, respectively. 

In this study, the soil organic matter content gradually decreased as the amount of AMD added to the soils rose. The soil organic matter content in the Taigu County soils decreased to 15.36 g/kg, which was equivalent to 16.2%, when the AMD volume to soil mass ratio was 8–10 mL/g. This decrease has also been observed in red soil systems. Tian et al. [42] reported that the soil total organic carbon content decreased by 30.41% when red soil was affected by acid rain and the pH was 2.50. The main reason for this may be as follows: the solubility of soil organic matter in the free state may increase as the soil pH decreases; and some organic-mineral compounds may be decomposed and destroyed under acidic conditions, which further increases soil organic matter leaching from soil systems. 

**Figure 5:**
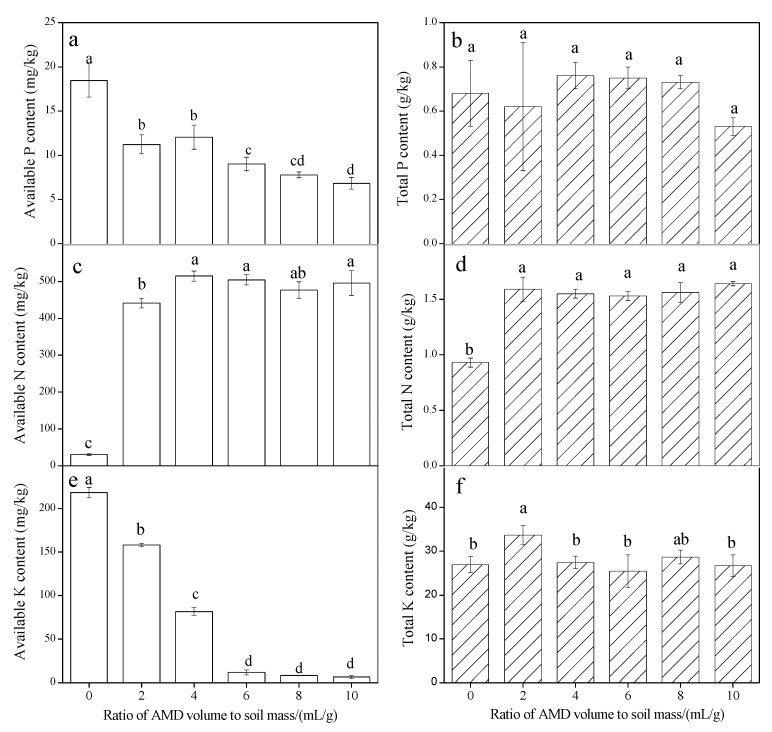
Available P (**a**), total P (**b**), available N (**c**), total N (**d**), available K(**e**), and total K (**f**) contents in AMD-polluted soil collected from Taigu County. Different letters on each column indicate that they are significantly different at *p* < 0.05.

Total N, total P, and total K, available N, available P, and available K contents in AMD-polluted soil collected from Taigu County are shown in Figure 5. The available P (Figure 5a) and available K (Figure 5e) in the soil collected from Taigu County gradually decreased as the amount of AMD added to the soil increased, whereas the total P (Figure 5b) and total K (Figure 5f) contents in the soil did not significantly decrease. For example, the available P and available K decreased by 63.0% and 97.1%, respectively, when the AMD volume to soil mass ratio was 10 mL/g. Mullins [43] reported that the P reacts with Fe to form insoluble Fe phosphates in acid solids. In this study, Fe^3+^ entered the soil systems along with the AMD and could have combined with the phosphate in the soil to form insoluble Fe-phosphate compounds. This could possibly be the main reason for the decrease in available P. When AMD was added to the soil, the extra H^+^ supplied by the AMD was absorbed onto the surfaces of soil particles, which would lead to a decrease in the negative charge of the soil surface. This would reduce the soil adsorption capacity for K^+^, which means that the available K would gradually leach out of the soil system. About 90–98% of total K in the soil is in unavailable structural or mineral forms [44]. In this study, the acidic soil environment (pH 2–7) caused by the addition of AMD was not enough to disintegrate the soil structure and promote the release of potassium because there was no significant change to total K content in AMD-affected soil (Figure 5f).

The available N and total N increased 14.4 times and 1.71 times, respectively compared with the initial soil when the AMD volume to soil mass ratio was 2 mL/g. However, they only increased to 16.1 times and 1.76 times, respectively, when the added AMD volume to soil mass ratio was 10 mL/g (Figure 5c,d). It is clear that available N and total N have no significant effect when the AMD volume to soil mass ratio increased from 2 mL/g to 10 mL/g. Previous studies have shown that low soil pHs caused by acid rain may restrict the adsorption of ammonium onto soil particles [45]. However, ammonium concentration in AMD was ~700 mg/L in this study. Therefore, the large amount of ammonium in AMD may increase the chance that ammonium comes into contact with soil particles, which means that available N and total N levels in AMD-affected soil are relatively stable. For example, when the AMD volume to soil mass ratio increased from 2 mL/g to 10 mL/g, the pH of the AMD-affected soil decreased from 7.65 to 3.10 but the available N and total N in the AMD-effected soil remained constant.

It can be concluded that the available N content in the soil will not decrease when AMD is added to soils, and that some of the nitrogen in the AMD will enter the soil in the form of ammonium, which would increase the available N and total N levels in AMD-polluted soil. In general, the loss of available P and available K from soils requires more attention than available N loss when AMD enters calcareous soils.

### 3.4. Effects of AMD on Heavy Metal Contents and Oxidation States in Calcareous Soil

**Figure 6:**
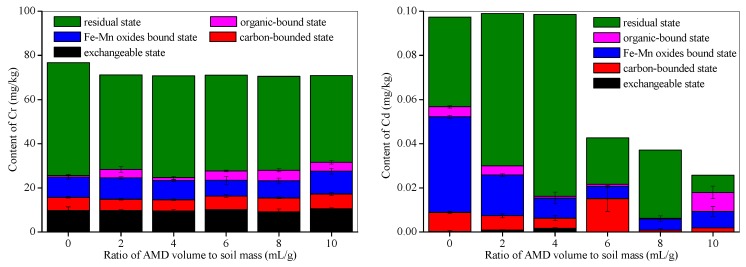
Cr and Cd contents and their oxidation state in AMD-polluted calcareous soil.

This study used Cr and Cd as representative heavy metals, and their contents and oxidation state in calcareous soil before and after AMD addition were investigated. The effect of AMD on heavy metal contents and their oxidation state in calcareous soil are shown in Figure 6.

The Cr contents in the soil slightly decreased when AMD was added. For example, the Cr concentration in the soil decreased from an initial 76.8 mg/kg to 70.8 mg/kg, when the AMD volume to soil mass ratio was 10 mL/g. The Cr contents that were in an exchangeable state, a carbon-bound state, Fe-Mn oxides bound state, an organic-bound state, or a residual state accounted for 12.7%, 7.8%, 11.9%, 0.83%, and 66.8%, respectively, of the total Cr content in the initial soil. However, the proportion of the total Cr in these oxidation state changed by 15.0%, 9.4%, 14.6%, 5.7%, and 55.4%, respectively, in AMD-polluted soil when the AMD volume to soil mass ratio was 10 mL/g. Further, the Figure 6 shows that the Cr proportion in the residual state decreases and that the Cr in an organic bound state increases when AMD was added to calcareous soil, but there was no significant change in the Cr proportions represented by the other states. Overall, adding AMD does not significantly increase Cr leaching out of the calcareous soil system. In iron rich sulfate systems, the Fe^3+^ ions tend to get hydrolyzed to ferrihydrite and schwertmannite at pHs higher than 5.5 or between 2.5–5.5 [46], and ferrihydrite and schwertmannite have good Cr adsorption properties [47,48]. The Cr adsorbed on the iron hydrolysis products, such as ferrihydrite and schwertmannite, may explain the low Cr leaching rate from AMD polluted calcareous soil. 

In contrast, as seen from Figure 6, a considerable amount of the Cd in the calcareous soil dissolved when the AMD volume to soil mass ratio was above 6 mL/g. The Cd concentrations in the soil decreased by 55.7%, 61.9%, and 73.2% when the AMD volume to soil mass ratio reached 6 mL/g, 8 mL/g, and 10 mL/g, respectively. The total amount of Cd in the soil did not change significantly when the AMD volume to soil mass ratio below 4 mL/g, and Cr in Fe-Mn oxides bound state tended to change to the residual state. When the results presented in Figure 2 and Figure 6 are compared, it can be seen that a soil pH below 3.5 can lead to significant dissolution of Cd from calcareous soil. Strobel et al. [49] found that the release of Cd from cultivated soils with an initial pH of 6.5 was controlled by the solution pH, and that twice as much Cd was released from soil at pH 3.9 than at pH 5.2. This suggests that high pH value of calcareous soils may reduce the Cd environmental risk caused by soil acidification, even though the adsorption efficiency of hydrolysis products, such as schwertmannite, for Cd is very weak [47]. In conclusion, the addition of AMD significantly accelerated the dissolution of Cd in soil compared to Cr. 

### 3.5. Effects of AMD-Polluted Calcareous Soil on Lolium Perenne L. Seed Germination 

**Figure 7:**
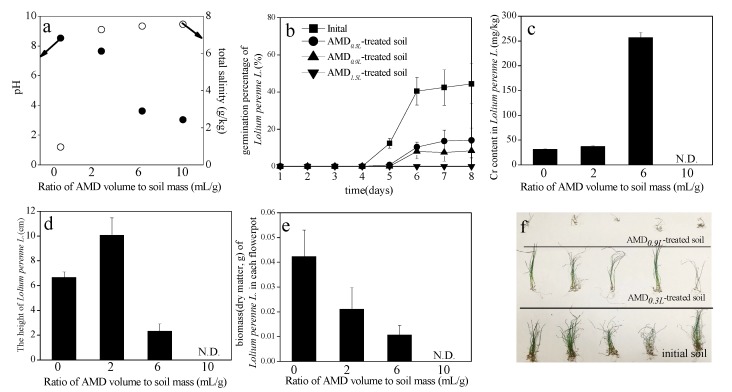
Effects of AMD-polluted calcareous soil on *Lolium perenne* L. seed germination efficiency. (**a**) pH and total salinity of the AMD-polluted soil; (**b**) *Lolium perenne* L. germination percentage for the different AMD-polluted soil treatments over time; (**c**) Cr content in *Lolium perenne* L. collected from the different AMD-polluted soil treatments; (**d**) heights of *Lolium perenne* L. collected from the different AMD-polluted soil treatments; (**e**) biomass (dry matter) of *Lolium perenne* L. collected from the different AMD-polluted soil treatments; (**f**) photographs of *Lolium perenne* L. collected from the different AMD-polluted soil treatments.

The pH and total salinity of AMD-polluted soils decreased from 7.64 and 7.28 g/kg, 3.62 and 7.46 g/kg, and 3.04 and 7.56 g/kg from an initial pH and salinity of 8.52 and 0.96 g/kg when the AMD volume to soil mass ratios were 2, 6, and 10 mL/g, respectively, after six months at 20–25 °C (Figure 7a). The change in *Lolium perenne* L. germination percentage over time is shown in Figure 7b. A low pH and a high salt content in the soil clearly affected seed germination [50,51]. Peerzada and Naeem [51] reported that the Cenchrus biflorus Roxb. germination percentage decreased from 97.5% to 12.5% as salinity stress increased from 0 mM to 200 mM sodium chloride, and that there was no germination > 200 mM. The germination percentage for C. biflorus Roxb seed was also significantly affected by pH levels and was between 27.5% and 92.5% at pH 5–8 [51]. In this study, *Lolium perenne* L. germination percentages on the eighth day were 44.4%, 14.0%, and 8.4% for the initial soil, the AMD_0.3L_-treated soil (AMD volume to soil mass ratio: 2 mL/g), and AMD_0.9L_-treated soil (AMD volume to soil mass ratio: 6 mL/g), respectively. However, the *Lolium perenne* L. failed to germinate after 8 days in the AMD_1.5L_-treated soil (AMD volume to soil mass: 10 mL/g). This suggested that when the AMD volume to soil mass ratio reached 10 mL/g, the soil conditions could not support *Lolium perenne* L. germination because the soil was too acidic (pH 3.04). Furthermore, the large amounts of salts released by the AMD (Fe^3+^, SO_4_^2−^, etc.) accumulated in the soil, which meant that the total soil salinity was 7.56 g/kg. The Cr contents in *Lolium perenne* L. collected from the different AMD-polluted soil treatments are shown in Figure 7c. It is clear that the addition of AMD promoted the adsorption of Cr by *Lolium perenne* L. However, the Cr contents in *Lolium perenne* L. reached 37.21 mg/kg and 256.85 mg/kg, which represents a 1.19 fold and 8.24 fold increase compared to the Cr content in *Lolium perenne* L. harvested from the original soil, when the AMD volume to soil mass ratio reached 2 mL/g and 6 mL/g, respectively. Although AMD inhibited *Lolium perenne* L. seed germination, the height of *Lolium perenne* L. harvested from AMD_0.3L_-treated soil was 1.51 times higher than the height of *Lolium perenne* L. harvested from the original soil (Figure 7d). However, the height of *Lolium perenne* L.harvested from AMD_0.9L_-treated soil was only 34.9% of that of one harvested from the original soil (Figure 7d). It is suggested that the available N (Figure 5c) introduced at certain AMD levels, such as 2 mL/g level, could increase *Lolium perenne* L. growth because pH (pH 7.64) did not dramatically change before and after the introduction of AMD. However, *Lolium perenne* L. growth was inhibited when excess AMD was applied, such as 6 mL/g, due to low pH (pH 3.62) of the AMD-polluted soil. When Figure 7a,e are compared, the results show that *Lolium perenne* L. biomass is more closely related to soil pH than soil total salinity. Zhu et al. [52] also indicated that Vallisneria natans L. biomass (dry matter) increased along with ammonium concentration when the ammonium in the sediment was less than 50 mg/kg. The average *Lolium perenne* L. biomasses (dry matter) in a flowerpot taken from the different treatments are shown in Figure 7e. The biomass of *Lolium perenne* L. harvested from the initial soil was two times more than *Lolium perenne* L. biomass harvested from AMD_0.3L_-treated soil and four times more than *Lolium perenne* L. biomass harvested from AMD_0.9L_-treated soil. However, the single *Lolium perenne* L. biomass harvested from the initial soil was just 56% of *Lolium perenne* L. biomass harvested from AMD_0.3L_-treated soil. This suggests that an AMD volume to soil mass ratio of 2 mL/g, reduced *Lolium perenne* L. germination, but increased its height and biomass. The physical states of the *Lolium perenne* L. collected from the different treatments is shown in Figure 7f.

In addition, it is noted that the *Lolium perenne* L. seed germination rate was significantly correlated with the pH and total salinity of AMD-polluted soil with the correlation coefficients were 0.835 and −0.936.

## 4. Conclusions and Prospects

When AMD contaminates calcareous soil, the total CaO and MgO contents in calcareous soil determines the acid buffering capacity of the soil against AMD. In calcareous soil, greater the CaO and MgO levels in the soil, higher the soil pH. Furthermore, more Fe^2+^ is transformed into Fe^3+^ in the soil system, more total Fe and SO_4_^2−^ are retained by the soil, and weaker the vertical migration capacity of the AMD major ions (H^+^, Fe, SO_4_^2−^) in the soil with high pH. 

When AMD was added to the soil, total P and K in the soil showed no obvious changes, but the available P and available K contents significantly decreased. The introduction of AMD increased the total N content in soil because the AMD contained considerable amounts of ammonia nitrogen, and even when the soil pH decreased to ~3.00, there was still no obvious leaching of available N from the soil. The introduction of AMD increased Cd and Cr leaching from the soil, but the leaching rate of Cd was higher than that of Cr. The addition of AMD decreased the *Lolium perenne* L. germination rate, and Cr accumulation in *Lolium perenne* L. increased when the AMD volume to soil mass ratio was 6 mL/g, and the pH and total salinity of the soil were 3.62 and 7.46 g/kg, respectively. However, the *Lolium perenne* L. could not germinate when the AMD volume to soil mass ratio was 10 mL/g, and the pH and total salinity of the soil were 3.04 and 7.56 g/kg, respectively. It is noted that the effect of cyclic redox conditions on the minerals in soil system may be very important in regulating the availability and immobility of a wide range of trace metals, metalloids, and nutrients [41], which we plan to focus in our future research.

This study has explored the behavior of the main pollutants (H^+^, total Fe, Fe^2+^, and SO_4_^2−^) in AMD when it was added at different rates to calcareous soil and found that the content of total calcium and magnesium oxide in soil is the main control factor of pollutants behavior. The influence of AMD on calcareous soil characteristics and the effect of AMD-contaminated soil on grass seed germination were also determined. According to this investigation, it is possible to understanding the hydrological /geochemical-behaviour of major ions of AMD in calcareous soil. The outcomes of this study also have a great significance in predicting plant growth behavior in AMD-polluted calcareous soil. It is noted that the findings of this study may have some limitations because the soil samples were collected from a specific area in Shanxi Province in China. 

Further studies need to be carried out to investigate the calcareous soil microbial activity and enzyme activity when the calcareous soil was polluted by AMD. 

## Figures and Tables

**Table 1:** Physical and chemical characteristics of the soil samples.

Soil Sample	pH	Organic Matter (g/kg d.w.)	Total N (g/kg d.w.)	Total P (g/kg d.w.)	Total K (g/kg d.w.)	Available N (mg/kg d.w.)	Available P (mg/kg d.w.)	Available K (mg/kg d.w.)	CaO (% d.w.)	MgO (% d.w.)	Physical Clay Content (<0.01 mm) (% d.w.)
Taigu County	8.52	18.32	0.93	0.68	26.94	30.71	18.45	218.23	4.17	2.25	19.87
Xiaoyi County	7.64	24.99	1.12	0.58	21.43	45.55	44.85	127.40	5.74	1.89	27.42
Xiangning County	8.62	19.15	1.05	0.08	19.42	51.77	7.08	153.37	4.76	1.96	39.66
Hejin County	8.37	26.59	0.80	0.20	16.92	37.93	17.57	316.13	11.36	2.87	19.46
Xixian County	8.36	16.96	1.05	0.56	18.67	37.32	43.22	90.83	7.28	2.24	25.58

Note: Physical clay is delineated according to Katschinski’s system of the former Soviet Union; d.w. indicates dry weight.

**Table 2:** Correlation analyses between soil pH reduction and soil property parameters when AMD (acid mine drainage) was added to the soil.

	CaO	CaO + MgO	MgO	Organic Matter	Clay Content	Available N	Available P	Available K	Total N	Total P	Total K
Soil pH reduction	−0.906 *	−0.913*	−0.847	−0.817	0.531	0.183	0.135	−0.812	0.752	0.356	0.505

Note: * indicates significant correlation at the *p* < 0.05 level.
